# Specific Composition of Lipid Phases Allows Retaining an Optimal Thylakoid Membrane Fluidity in Plant Response to Low-Temperature Treatment

**DOI:** 10.3389/fpls.2020.00723

**Published:** 2020-06-05

**Authors:** Radosław Mazur, Katarzyna Gieczewska, Łucja Kowalewska, Anna Kuta, Małgorzata Proboszcz, Wieslaw I. Gruszecki, Agnieszka Mostowska, Maciej Garstka

**Affiliations:** ^1^Department of Metabolic Regulation, Faculty of Biology, Institute of Biochemistry, University of Warsaw, Warsaw, Poland; ^2^Department of Plant Anatomy and Cytology, Faculty of Biology, Institute of Plant Experimental Biology and Biotechnology, University of Warsaw, Warsaw, Poland; ^3^Department of Biophysics, Institute of Physics, Maria Curie-Skłodowska University, Lublin, Poland

**Keywords:** photosynthesis, chilling stress, thylakoid membranes, membrane fluidity, lipid composition, pea, bean, LHCII phosphorylation

## Abstract

Thylakoid membranes isolated from leaves of two plant species, the chilling tolerant (CT) pea and chilling sensitive (CS) runner bean, were assessed for the composition of lipids, carotenoids as well as for the arrangement of photosynthetic complexes. The response to stress conditions was investigated in dark-chilled and subsequently photo-activated detached leaves of pea and bean. Thylakoids of both species have a similar level of monogalactosyldiacylglycerol (MGDG) and digalactosyldiacylglycerol (DGDG), but different sulfoquinovosyldiacylglycerol to phosphatidylglycerol (PG) ratio. In pea thylakoid fraction, the MGDG, DGDG and PG, have a higher double bond index (DBI), whereas bean thylakoids contain higher levels of high melting point PG. Furthermore, the lutein to the β-carotene ratio is higher in bean thylakoids. Smaller protein/lipid ratio in pea than in bean thylakoids suggests different lipid-protein interactions in both species. The differences between species are also reflected by the course of temperature-dependent plots of chlorophyll fluorescence pointing various temperatures of the lipid phase transitions of pea and bean thylakoids. Our results showed higher fluidity of the thylakoid membrane network in pea than in bean in optimal temperature conditions. Dark-chilling decreases the photochemical activity and induces significant degradation of MGDG in bean but not in pea leaves. Similarly, substantial changes in the arrangement of photosynthetic complexes with increase in LHCII phosphorylation and disturbances of the thylakoid structure take place in bean thylakoids only. Changes in the physical properties of bean thylakoids are manifested by the conversion of a three-phase temperature-dependent plot to a one-phase plot. Subsequent photo-activation of chilled bean leaves caused a partial restoration of the photochemistry and of membrane physical properties, but not of the photosynthetic complexes arrangement nor the thylakoid network structure. Summarizing, the composition of the thylakoid lipid matrix of CT pea allows retaining the optimal fluidity of its chloroplast membranes under low temperatures. In contrast, the fluidity of CS bean thylakoids is drastically changed, leading to the reorganization of the supramolecular structure of the photosynthetic complexes and finally results in structural remodeling of the CS bean thylakoid network.

## Introduction

Thylakoid membranes in chloroplasts of higher plants are assemblies of chlorophyll-protein (CP) complexes and lipids, organized into two distinct domains: stacked membranes called grana and unstacked stroma thylakoids (Ruban and Johnson, 2015). CP complexes are organized hierarchically in supercomplexes and megacomplexes, and are spatially segregated (Kouřil et al., 2012). Photosystem II (PSII) with light-harvesting complexes (LHCII) is organized in LHCII-PSII supercomplexes and localized in the grana, whereas the LHCI-PSI supercomplexes consist of Photosystem I (PSI) and its antenna (LHCI), which are localized in unstacked thylakoids (Danielsson et al., 2004, 2006; Daum et al., 2010; Koochak et al., 2019). Dynamic changes in the arrangement of CP complexes and grana structure play a crucial role in the regulation of photosynthesis in response to environmental conditions (Wood et al., 2018; Johnson and Wientjes, 2019).

The lipid phase of thylakoids is formed by monogalactosyldiacylglycerol (MGDG) that accounts for about 50% of the total lipid content, digalactosyldiacylglycerol (DGDG) (∼30%), sulfoquinovosyldiacylglycerol (SQDG) (∼5–12%), and phosphatidylglycerol (PG) (∼5–12%) (Pali et al., 2003). The MGDG, the lipid forming inverted hexagonal phase, is linked with the dynamic polymorphism of lipids in thylakoid membranes, where the main bilayer phase coexists with minor non-bilayer domains (Garab et al., 2017). The formation of large lamellar structures is related to the presence of integral membrane proteins, mainly LHCII, which inhibits the formation of a non-bilayer structure (Simidjiev et al., 2000; Janik et al., 2013; Kowalewska et al., 2016). On the other hand, the MGDG protects the LHCII against unfolding (Seiwert et al., 2017). Furthermore, specific lipids bound inside the PSI, PSII or LHCII enable the functional conformation and photochemical activity of CP complexes (Jones, 2007; Domonkos et al., 2008; Mizusawa and Wada, 2012; Kobayashi et al., 2017). Membrane fluidity increases with the degree of desaturation of lipid acyl chains and with the content of β-carotene, whereas the presence of xanthophylls and α-tocopherol incorporated into bilayer leads to an increase of membrane rigidity (Pali et al., 2003; Gruszecki and Strzalka, 2005; Szilagyi et al., 2008). The ratio of lipids to proteins in thylakoids is estimated to be roughly 0.3 (Koochak et al., 2019); therefore, the membrane dynamics might be substantially modulated by lipid-protein interactions (Pali et al., 2003).

The lipid composition of thylakoids is an important factor determining the stabilization of the photosynthetic complexes under low temperature. An increase of the level of desaturated lipids is correlated with the increase of plant resistance to chilling conditions, as was described for many cold-adapted plants (Zheng et al., 2016; Kenchanmane Raju et al., 2018), as well as for plants with elevated (Orlova et al., 2003; Khodakovskaya et al., 2006) or decreased (Ivanov et al., 2012; Chen and Thelen, 2013) acyl-lipid desaturases. A decrease of MGDG/DGDG ratio and the level of PG induced by low temperature is generally present but varies depending on the plant species (Zheng et al., 2016; Skupien et al., 2017; Kenchanmane Raju et al., 2018).

The response of plant species to low temperature is also related to their evolutionary background. The chilling-sensitive plants (CS), susceptible to temperatures below 12°C, mostly originated from subtropical areas, whereas the chilling-tolerant (CT) ones, resistant to low but non-freezing temperatures, evolved in temperate climate (Garstka et al., 2005, 2007). In many cases, the chilling-stress is studied using the dark-chilling model in which observed effects are due mainly to the low temperature alone; chilling in the light may cause photo-inhibition of both photosystems and strong oxidative stress (Garstka et al., 2007; Mazur et al., 2018).

Low temperature induces up-regulation of genes involved in PG and galactolipid synthesis and their remodeling both in CT and CS plants (Gu et al., 2017; Marla et al., 2017; Kenchanmane Raju et al., 2018). The expression of *type 2* plastid-localized lipoxygenase, an enzyme catalyzing oxygenation of polyunsaturated fatty acids, is noted in CS plants (Mazur et al., 2018). In many CS plants, dark-chilling leads to the accumulation of free fatty acids (FFA) due to the high activity of galactolipase under these conditions (Kaniuga, 2008). Furthermore, a higher proportion of motionally restricted lipids, localized in the boundary phase of CP complexes, is registered in CS compared with CT plants, indicating the role of lipid-protein interaction in chilling response (Li et al., 1990).

Apart from the lipid phases composition, plant response to low temperature is also considered in the context of CP complexes arrangement. A decrease in PSII activity in detached leaves of CS plants chilled in the dark is associated with the release of manganese from the oxygen-evolving complex of PSII (Kaniuga, 2008) and with the destabilization of PSII extrinsic proteins (Shen et al., 1990; Garstka and Kaniuga, 1991; Higuchi et al., 2003). Dark-chilling induces rearrangements of CP supercomplexes – LHCI-PSI and LHCII trimers leading to the disturbance of thylakoids structure in CS bean and tomato (Garstka et al., 2005, 2007).

In the present paper, we studied the effect of low temperature on chloroplasts and thylakoids isolated from dark-chilled and subsequently photo-activated detached leaves of CT garden pea and CS runner bean. We investigated whether the differences in the composition of lipid phases, arrangement of CP complexes and their phosphorylation determine the response of these species to low temperature. Complex analyses of lipid and carotenoid compositions of pea and bean thylakoids were performed. Moreover, the structural arrangements of CP complexes were examined by time-resolved, temperature-dependent and low-temperature chlorophyll *a* (Chl *a*) fluorescence, as well as by Fourier-transform infrared spectroscopy (FTIR). Data were complemented by imaging the chloroplast structure by confocal laser scanning (CLSM) and transmission electron microscopy (TEM). The protein phosphorylation was studied using the electrophoretic technique. The relations between microscopic, biochemical and biophysical data were discussed in detail regarding lipid-protein interactions and reversible protein phosphorylation.

We found that the pea and bean thylakoid membranes differ in lipid and carotenoid composition, lipid desaturation level, protein/lipid ratio and arrangement of CP complexes. These features significantly influence the physical properties of thylakoid membranes. The dark-chilling treatment does not influence the physical properties of pea thylakoids and high fluidity of their membranes is preserved at low temperatures. On the contrary, more rigid bean thylakoid membranes change significantly under dark-chilling conditions leading to the reorganization of the supramolecular structure of photosynthetic complexes and structural remodeling of the CS bean thylakoid network.

## Materials and Methods

### Plant Growth and Chilling Treatment

Runner bean (*Phaseolus coccineus* L. cv. Eureka) and garden pea (*Pisum sativum* L. cv. Demon) plants (both from PlantiCo Zielonki, 05-082 Babice Stare, Poland) were grown in 3 L perlite-containing pots in a climate-controlled room (21°C day/18°C night) at a photosynthetic active radiation (PAR) of 200 μmol photons m^–2^ s^–1^ during a 16 h photoperiod and relative humidity of 60–70%. Fully expanded primary leaves of 10 day-old bean and 3rd–4th leaves of 20 day-old pea were harvested 30 min after the light was switched on. For the control samples the thylakoids were immediately isolated from harvested leaves or intact leaves were used for *in vivo* fluorescence measurements. For dark-chilling treatment, the detached leaves of bean and green parts of pea were placed in Dewar flasks (4°C, 100% relative humidity) for 5 days. For photo-activation, dark-chilled leaves/green parts were placed on a water layer in a transparent plastic dish at 21°C with PAR of 200 μmol of photons m^–2^ s^–1^ and optimal humidity for 3 h.

### Preparation of Chloroplasts and Thylakoid Membranes

Intact chloroplasts and thylakoid membranes were isolated by homogenization of pea and bean leaves in a buffered isotonic medium and subsequent centrifugation as described previously (Rumak et al., 2012). The concentration of chlorophyll was quantified spectrophotometrically after extraction with 80% (v:v) acetone (Rumak et al., 2012).

### *In vivo* Chlorophyll *a* Fluorescence and P700 Measurements

Chlorophyll *a* fluorescence and P700 absorption changes were measured *in vivo* by the Pulse-Amplitude-Modulation approach using the Dual-PAM 100 (Heinz Walz GmbH, Effeltrich, Germany). Before all measurements, plants were dark-adapted for 30 min. Minimal (F_0_) and maximal (F_M_) fluorescence were measured by applying red light pulse with intensity below 1 μmol photons m^–2^ s^–1^ and 90 ms red light saturation pulse with 20,000 μmol photons m^–2^ s^–1^, respectively. Simultaneously fast kinetics curves were measured during 160 ms and 10 μs data collection intervals.

Kinetics of P700 oxidation were measured with the help of far-red illumination. Dark-adapted leaves were pre-illuminated for 5 min with far-red light of intensity ∼250 μmol photons m^–2^ s^–1^. Next, the single turnover (ST) flash (50 μs, 10,000 μmol photons m^–2^ s^–1^) and after 10 s the multiple turnover (MT) flash (300 ms, 10,000 μmol photons m^–2^ s^–1^) were applied. The first 4 s after MT flash of P700 signals were analyzed; the amplitude of the P700 signals was normalized to 1.

The capacity of intersystem electron carrier pool P700 was measured by a similar approach, as described above. The dark-adapted leaves were illuminated with far-red light of intensity ∼50 μmol photons m^–2^ s^–1^ during 60 s. Next, the ST (50 μs) and MT (50 ms) flashes were applied with 10 s between them, and the P700 signal was recorded 120 s after applying MT. The P700 reduction areas induced by ST and MT were used for calculation of the capacity of the intersystem electron carrier pool, basing on the assumption that MT flash fills up the intersystem carriers pool (Asada et al., 1992).

Non-photochemical plastoquinone (PQ) reduction was measured according to (Shikanai et al., 1998) with some modifications. Dark-adapted leaves were illuminated by red actinic light of the intensity of 750 μmol photons m^–2^ s^–1^ for 5 min, followed by 3 min in darkness. The fluorescence signal was measured by weak modulated blue light with a 5 ms interval.

### Determination of Temperature-Dependent Chl *a* Fluorescence of Thylakoid Membranes

The temperature-dependent changes in Chl *a* fluorescence were analyzed according to Gruszecki et al. (1999) with some modifications. The intensity of fluorescence emission was determined with the Shimadzu RF-5301PC spectrofluorimeter with 3 and 10 nm spectral resolution for excitation and emission, respectively. The thylakoid suspension (12 μg Chl mL^–1^) placed in a sealed quartz cuvette (10 mm optical path length) was magnetically stirred to prevent settling. The sample was initially cooled to 1°C and then the temperature was increased gradually from 1 to 40°C with 1°C interval. During sample stabilization at particular temperature the sample was kept in darkness for 1 min, and then the shutter of the spectrofluorimeter was opened for 20 s only. The intensities of exctitation light were 40 and 100 μmol photons m^–2^ s^–1^ for 412 and 470 nm, respectively. The fluorescence intensity at two excitation/emission wavelengths (412/680 nm; 470/680 nm) was recorded simultaneously, 40 points for each degree at 0.5 s interval, but average values were calculated only for the last 20 points, where the fluorescence level was stable.

### Low-Temperature Fluorescence Measurements

Steady-state fluorescence emission spectra of chlorophyll at low temperature (77 K) were recorded using the modified Shimadzu RF-5301PC spectrofluorimeter with the emission and excitation beams guided through the light pipes. Thylakoid membranes diluted to chlorophyll concentration of 10 μg/mL were placed in a polytetrafluoroethylene cuvette and submerged in liquid nitrogen. The excitation wavelength was set at 412 and 470 nm, excitation and emission slits at 5 nm, and scans were taken in the range of 600–800 nm through the LP600 emission filter.

### Fourier-Transform Infrared Spectroscopy (FTIR) Measurements

Thylakoid membranes isolated from control, chilled, and photoactivated plants were resuspended in a ^2^H_2_O-based 20 mM Hepes–NaOH (pH 7.0) buffer containing 330 mM sorbitol, 15 mM NaCl and 4 mM MgCl_2_ and then centrifuged at 5000 *g* for 5 min at 4°C. This step was repeated three times to replace the H_2_O based buffers with the ^2^H_2_O ones. Fourier-transform infrared (FTIR) spectra were recorded with a Bruker Vector 33 spectrometer equipped with a horizontal attenuated total reflection (ATR) crystal as described previously (Rumak et al., 2010).

### Extraction and Analysis of Polar Lipids

Thylakoid membranes containing 0.8 mg of chlorophyll were dissolved in 6 mL of chloroform: methanol 2:1 (v:v) mixture. Total polar lipid extraction was performed as described in Skupien et al. (2017). Extracted lipids were separated using the Waters 600 HPLC system connected with ZQ 2000 mass spectrometer (Waters). The samples were injected (20 μL) into a Discovery^TM^ RP Amide C16 (5.0 μm, 180 Å, 2.1 × 150 mm) column equilibrated in 20% of solvent A (water) and 80% of solvent B (methanol: acetonitrile 7:3 (v:v). Elution was carried out with a constant flow rate of 0.3 mL/min with linearly increasing solvent B to 90% for 20 min. Next, a stepped linear gradient of solvents B and C (2-propanol) was distributed as follows: 90–100% B in the 20–70 min; 100% B in 71–80 min; 100–30% B and 0–70% C in 80–100 min. The column was re-equilibrated in 30% of A and 70% of B for 20 min before the next injection. The quality of sample separation was monitored by an absorption detector at 210 and 436 nm. Mass spectra between 500 and 1000 m/z were recorded in a positive and negative mode using the ZQ 2000 single quadrupole mass spectrometer with an electrospray ion source. The capillary and cone voltages were the same for the positive and negative ionization and were set at 4 kV and 30 V, respectively. Specific lipids were assigned to molecular masses by a comparison of the collected mass spectra with the standards and with the literature data (Skupien et al., 2017). Up to 35 molecular species of four classes of lipids (MGDG, DGDG, SQDG, and PG) were identified. Quantitative analysis was performed on the basis of areas under the spectrum calculated using the MassLynx v3.5 software (Waters) and results were presented as a relative composition of lipid classes. The double-bond index (DBI) and acyl chain length (ACL) were calculated as a sum of percentage participation of the total number of double bonds (N) or the total number of carbons (n) in the two fatty acid chains of each lipid molecular species or of all identified lipids, according to equation DBI(ACL) = Σ[N(n) × % lipid])/100 (Zheng et al., 2016).

### Extraction and Analysis of Carotenoids

Pigments, including carotenoids, were extracted as described earlier (Szalonek et al., 2015). Extracted pigments were separated using Waters 600 HPLC system (Waters). The samples were injected (20 μL) into an Atlantis^TM^ dC18 (3 μm, 100 Å, 3.0 × 150 mm) analytical column equilibrated in 100% of solvent A (water : methanol 1:9, v:v). The column was eluted at 25°C at a constant flow rate of 0.225 mL/min with 100% of solvent A for 10 min. Next the stepped linear gradient of buffer B (methanol : 2-propanol : hexane 2:1:1, v:v:v) was distributed as follows: 0–20% of B in the 10–42 min (flow rate 0.225–0.32 mL/min); 20–70% of B in 42–92 min and 70–100% of B in 92–120 min (flow rate 0.32 mL/min); and held for 10 min more at 100% of B with increasing flow rate to 0.5 mL/min. In the next 5 min the concentration of solvent B was decreased to 0% and the column was equilibrated for 15 more min (flow rate 0.5 mL/min) before the next injection. The separation of samples was monitored by an absorption detector at 436 and 652 nm. For quantification of carotenoids, chromatogram at 436 nm was integrated using MassLynx 3.5 software (Waters) and results were presented as a relative contribution of specific carotenoids in the total carotenoid fraction.

### Electrophoretic Techniques

Thylakoid membrane proteins were separated by the standard SDS-PAGE technique using 14% polyacrylamide (with no addition of urea) resolving gels. Phospho-protein and protein staining were performed using the ProQ^®^-Diamond and SYPRO^®^ Ruby according to the manufacturer (Invitrogen^TM^, cat. no MPM33305) protocol. Briefly, after fixation in 50% methanol, 10% acetic acid gels were washed (3 × 10 min) in ultrapure water and stained in the ProQ^®^-Diamond during 90 min in the dark. Stained gels were placed in a destaining solution (20% acetonitrile, 50 mM sodium acetate pH 4.0) for 3 × 30 min in the dark. After washing (2 × 5 min, ultrapure water) gels were scanned using the Typhoon FLA9000 laser scanner (Amersham Biosciences). The excitation source was set to 532 nm and fluorescence was detected through a 560 nm long-pass emission filter. Scanned gels were washed in 50% methanol, 10% acetic acid during 30 min and stained in the SYPRO^®^ Ruby overnight. Then, gels were washed in 10% methanol, 7% acetic acid during 30 min following 2 × 5 min in ultrapure water. The SYPRO^®^ Ruby fluorescence was detected using the Typhoon FLA9000 scanner with a 473 nm laser source and a 560 nm longpass emission filter. The other parameters were the same as for the Pro-Q^®^ Diamond. Relative band intensities were quantified using the Quantity One software (Bio-Rad, United States). Bands of selected phosphoproteins were analyzed by densitometry; selected band pixel intensities of ProQ^®^ Diamond signal were divided by corresponding bands intensities of SYPRO^®^ Ruby signal to eliminate possible unequal protein content.

### Microscopy Techniques

For Transmission Electron Microscopy (TEM) samples of ca. 3 mm^2^ were cut from middle parts of the leaves and prepared as described previously (Kowalewska et al., 2016). The 70 nm thick sections were examined with a JEM 1400 electron microscope (Jeol, Japan).

For Confocal Laser Scanning Microscopy (CLSM) isolated intact chloroplasts were suspended in 20 mM HEPES-NaOH buffer (pH 7.5) containing 330 mM sorbitol, 6% (v/v) glycerol, 15 mM NaCl, 4 mM MgCl_2_, and 30 μM DCMU to a final chlorophyll concentration of 30 μg mL^–1^. After 10 min of incubation in the dark and on ice, the suspension was immobilized on a microscope glass covered with poly-L-lysine. Chloroplast images were taken using the Zeiss LSM 510 confocal laser scanning fluorescence microscope as described previously (Rumak et al., 2012). The collected data sets were deconvolved using the AutoQuant X3 software (Media Cybernetics Inc., United States).

### Statistical Analysis

The statistical significance of differences between species and experimental conditions was verified by one-way ANOVA with *post hoc* Tukey test at *p* = 0.05. The number of repetitions of specific experiments are given in the figure legends and table footnotes.

## Results

### Modification of Photochemical Activity by Dark-Chilling Conditions

**FIGURE 1:**
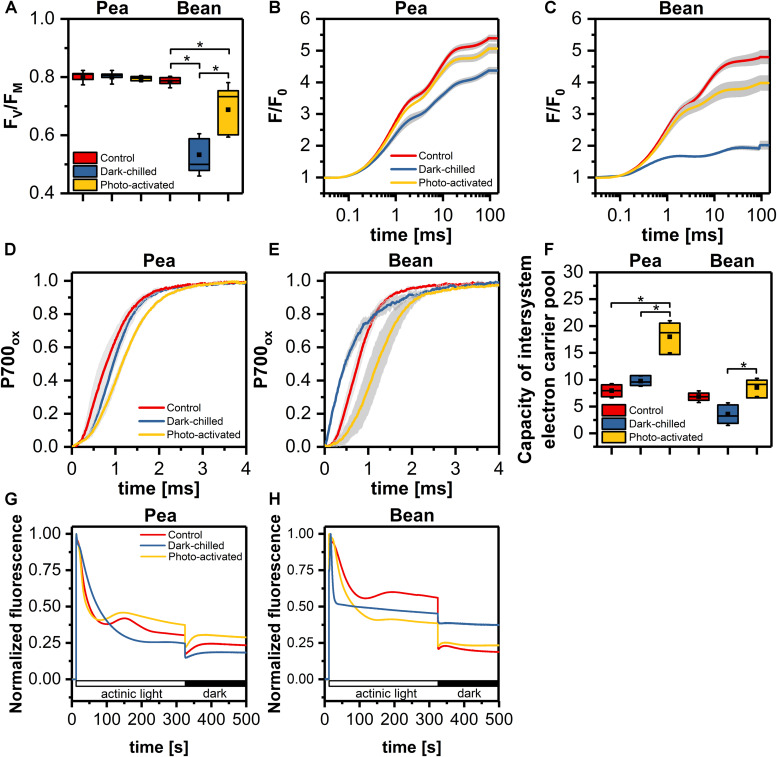
The efficiency of photosynthetic light reactions of pea and bean plants in control, dark-chilled, and photo-activated conditions **(A)** maximal quantum yield of PSII (F_V_/F_M_); **(B,C)** Chlorophyll *a* fluorescence fast induction curves of pea **(B)** and bean **(C)** leaves; **(D,E)** kinetics of P700 oxidation in pea **(D)** and bean **(E)** leaves; **(F)** capacity of intersystem electron carrier pool; **(G,H)** analysis of non-photochemical reduction of plastoquinone pool in pea **(G)** and bean **(H)**. The data are mean values ± SD from three independent experiments; pairs of results marked with an asterisk differ significantly at *p* = 0.05.

Detailed analysis of photochemical parameters related to the photochemical activity of both photosystems of pea and bean is presented in Figure 1. In pea, the maximal quantum yield of PSII (F_V_/F_M_) was stable in all experimental conditions (Figure 1A), while in bean, after a dark-chilling high decrease of F_V_/F_M_ value was observed with partial recovery after photo-activation (Figure 1A). The fast fluorescence induction curves (Figures 1B,C) showed that in pea there was some decrease of fluorescence intensity after dark-chilling comparing to control leaves, however, subsequent photo-activation led to almost complete recovery (Figure 1B). In bean, a decrease of fluorescence in dark-chilling leaves was much more pronounced and photo-activation resulted in weaker recovery comparing to pea (Figure 1C).

In order to determine photochemical efficiency downstream PSII, we examined the P700 oxidation kinetics and capacity of the intersystem electron carrier pool (Figures 1D–F). In pea, the P700 oxidation was slightly slower after dark-chilling and after subsequent photo-activation this effect was even more visible (Figure 1D). In contrast, in bean the dark-chilling caused an acceleration of the P700 oxidation kinetics comparing to control conditions (Figure 1E), however, after photo-activation, the P700 oxidation slowed down compared to the dark-chilled and control conditions (Figure 1E). Estimation of the intersystem electron carrier pool showed a substantial increase in the carrier pool in pea after photo-activation of dark-chilled samples (Figure 1F). In contrast, in bean, the carrier pool decreased after dark-chilling, while photo-activation induced recovery to the values observed in control leaves (Figure 1F).

To determine the activity of alternative electron routes, we measured non-photochemical reduction of PQ by changes in Chl *a* fluorescence in the presence of actinic light and the dark (Shikanai et al., 1998). The detection of reduced PQ in the darkness indicates the activity of Ndh and PGR5/PGRL1 dependent electron transport in thylakoids. The non-photochemical PQ reduction in control conditions was more effective in pea than in bean (Figures 1G,H). In pea, the dark-chilling induce a decrease of non-photochemical PQ reduction and after photo-activation the full recovery was observed (Figure 1G). In bean, the dark-chilling caused complete inactivation of PQ reduction in the dark, while photo-activation, similarly to pea, led to full recovery (Figure 1G).

### Analysis of Physical Properties of Thylakoid Membranes

**FIGURE 2:**
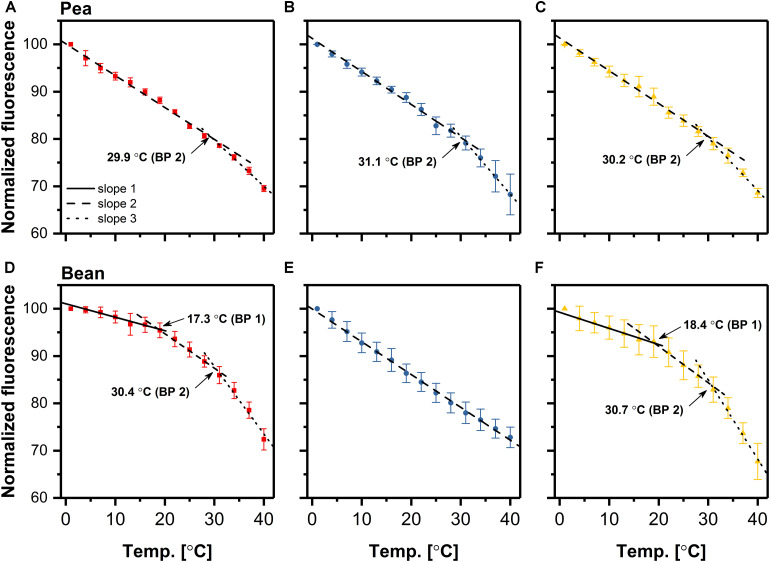
Temperature-dependent plots of the rate of chlorophyll fluorescence decrease in pea **(A–C)** and bean **(D–F)** thylakoids isolated from control **(A,D)**, dark-chilled **(B,E)** and subsequent photo-activated **(C,F)** leaves. Fluorescence emission at 680 nm was excited at 470 nm. The data are mean values ± SD for 3–5 independent experiments. The values for breakpoints (BP) were calculated by linear regression independently for each experiment. For a clear presentation of plots, two out of every three consecutive points were omitted.

We estimated the relationship between supramolecular membrane arrangement and the photochemical activity, via the analysis of temperature dependence of the steady-state Chl *a* fluorescence emission of thylakoids isolated from control, chilled and photo-activated leaves. The fluorescence emission at 680 nm was recorded simultaneously at two excitation (ex) wavelengths, allowing preferential excitation of Chl *a* (412 nm) and Chl *b* (470 nm). Fluorescence emission patterns recorded at both excitations (Figure 2 and Supplementary Figure 1), indicated that both core (ex 412 nm) and antenna (ex 470 nm) complexes revealed similar responses under the applied experimental conditions.

The intensity of fluorescence decreases with the increase of the temperature. In the case of control pea thylakoids, the linear regression analysis of the plots revealed, that the decrease of Chl *a* fluorescence, excited at 470 nm, exhibited two distinct linear phases (Figure 2A), in which the breakpoint between these phases was estimated to be roughly 30°C. The gradient of the temperature-dependent plot below this temperature was 40% smaller than for the phase between 30 and 40°C (Supplementary Table 1). The temperature-dependent plot of control bean thylakoids was more complex; three independent phases with two breakpoints were noted (Figure 2D). In the first phase, up to about 18°C, the rate of fluorescence decrease was almost temperature independent. The slope of the next phase, between 18 and 30°C was three times larger as compared to the first phase. The gradient in 18–30°C phase was similar to the 1–30°C phase in pea thylakoids (Supplementary Table 1). The third phase for control bean thylakoids, above 30°C, revealed a more rapid decrease of the Chl *a* fluorescence, with a 7-fold higher slope than in the first phase in bean, and noticeably higher than for the 30–40°C phase in pea thylakoids (Supplementary Table 1).

The temperature-dependent plots of fluorescence decrease for thylakoids isolated from dark-chilled and subsequently photo-activated pea revealed similar behavior to the control (Figures 2B,C), except for a small increase of slopes in second phases (Supplementary Table 1). These observations suggest a stable interaction between CP complexes in pea thylakoids despite applied temperature conditions. On the contrary, the temperature-dependent plot for thylakoids isolated from dark-chilled bean leaves were significantly different from the control ones, and had a single-phase dependence on the incubation temperature (Figure 2E); the slope was similar to that of the 1–30°C and 18–30°C phase registered in control pea and bean thylakoids, respectively (Supplementary Table 1). Lack of breakpoint indicated an increase of uniform interactions between CP complexes inside thylakoids, which allow the quench of the Chl *a* fluorescence at the same activation energy (Wentworth et al., 2003). Subsequent photo-activation of bean leaves restored a three-phase behavior of the temperature-dependent plot (Figure 2F) with similar breakpoints and the appropriate rate of fluorescence decrease (Supplementary Table 1), suggesting reconstruction of the initial interactions between CP complexes.

### Analysis of Photosynthetic Complexes Arrangement by Low-Temperature Fluorescence

The relative contribution of specific complexes to the normalized fluorescence emission spectra at 77 K was investigated in thylakoids isolated from control and stressed leaves. The typical spectrum consists of three main bands: (i) at around 683 nm, corresponding to emission from both trimers and monomers of LHCII, (ii) at around 693 nm originated from PSII core, (iii) at 734 nm related mainly to the PSI-LHCI.

**FIGURE 3:**
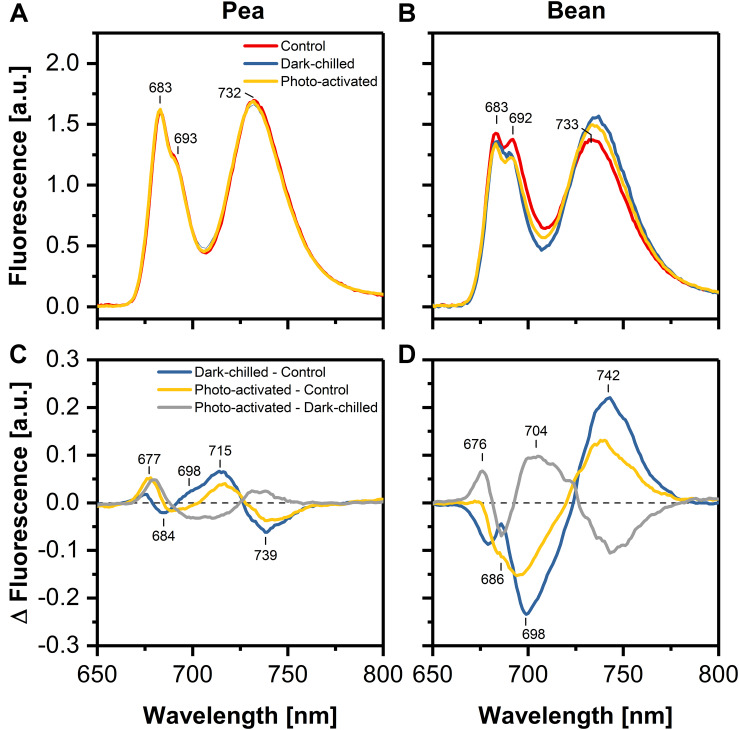
Effect of dark-chilling and subsequent photo-activation on fluorescence emission spectra (Ex 470 nm) at 77 K of isolated thylakoids from control (red), dark-chilled (blue) and subsequently photo-activated (orange) leaves of pea **(A)** and bean **(B)**, respectively. Lower panels present the corresponding difference fluorescence spectra for pea **(C)** and bean **(D)**. The spectra **(A,B)** were normalized to the area of 100 under the spectrum, and the arithmetic differences **(C,D)** between them were calculated. The presented spectra are representative of 3–5 independent experiments.

The chlorophyll fluorescence was excited at 412 nm (Chl *a*) (Supplementary Figure 2) and 470 nm (Chl *b*) (Figure 3), and difference spectra revealed similar shapes independent on the excitation wavelength. The ratio of fluorescence at 683–693 nm was estimated to 1.3 and 1.0 in pea and bean thylakoids, respectively. These data indicated a larger abundance of LHCII connected with PSII in pea compared with bean thylakoids.

The difference spectrum for thylakoids from dark-chilling pea leaves – minus – control showed a slight increase in the emission at around 698 and 715 nm accompanied by a simultaneous decrease of fluorescence around 739 nm. The subsequent photo-activation of leaves led to a partial recovery to the values observed in control conditions (Figure 3C).

Dark-chilling of bean leaves led to a decrease of emission at 686 and 698 nm related to the fluorescence emission from LHCII and PSII core complexes (Figure 3D). Furthermore, the difference spectrum exhibited a positive band at around 742 nm due to the emission from LHCI/PSI complexes. The intensities of these bands decreased upon photo-activation, which suggested a partial recovery of CP complexes organization during photo-activation. However, the difference spectrum calculated from the emission spectra for thylakoids isolated from the photo-activated and dark-chilled leaves revealed a positive band around 704 nm, indicating the formation of LHCII aggregates (Figure 3D).

### The Structural Relationship Between Lipids and Proteins in Pea and Bean Thylakoid Membranes

**FIGURE 4:**
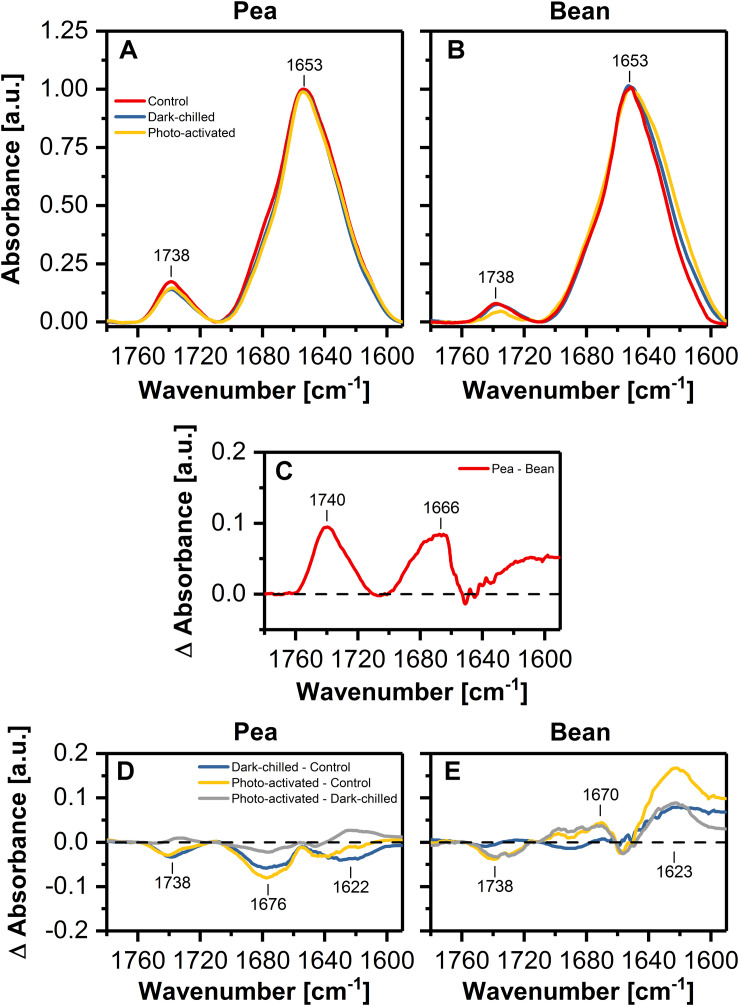
Normalized (at 1650 cm^–1^ to one) infrared absorption spectra in the Amide I and ester C=O regions recorded from pea **(A)** and bean **(B)** thylakoids isolated from control (red), dark-chilled (blue) and subsequently photo-activated (orange) leaves. **(C)** Presents the difference spectrum between pea and bean control spectra. Lower panels show the difference spectra between control and stressed thylakoids for pea **(D)** and bean **(E)**, respectively. The presented spectra are representative of 3 independent experiments.

The FTIR spectroscopy is a useful method to analyze the relationships between lipids and proteins, as well as the changes in the protein secondary structure. The band between 1760 and 1710 cm^–1^ is related to a ester C=O vibration; a bond originating exclusively from lipids, whereas Amide I region (1700–1580 cm^–1^) corresponds to the vibration of the peptide bond carbonyl group. The relative ratio of these band intensities, in spectrum normalized at 1650 cm^–1^, reflects the relative lipid to protein ratio (Szalontai et al., 2000; Rumak et al., 2010; Kovacs et al., 2019). In the case of our study, the calculated protein/lipid ratios (Figures 4A,B) for control pea and bean thylakoids were estimated to 9.46 ± 0.57 and 25.89 ± 1.96, respectively.

The pea – minus – bean FTIR difference spectrum revealed a positive band in the C=O vibration region, and additionally, bands related to the protein structure (Figure 4C). The Amide I region can be divided into several individual components attributed to different secondary structures of proteins – the region around 1650 cm^–1^ representing a protein α-helical structure and the region around 1690 cm^–1^ attributed to antiparallel β-sheet structures, probably formed by hydrogen bonds between the polar loops of thylakoid proteins localized outside the membrane. Additionally, the region around 1620 cm^–1^ can be assigned to a parallel β-structure associated with the formation of hydrogen bonds between α-helices of neighboring proteins in the lateral planes of membranes (Rumak et al., 2010; Janik et al., 2013). The pea – minus – bean thylakoid difference spectrum of Amid I region revealed noticeable differences in the regions attributed to the formation of both pseudo-β-structures formed between neighboring proteins in both planes of membranes (Figure 4C), suggesting higher interactions between proteins in pea than in bean thylakoids.

The difference FTIR spectrum for thylakoids of dark-chilled pea leaves (dark-chilled – minus – control) showed a slight decrease in the band assigned to lipids and in the band corresponding to the interactions between neighboring membrane proteins. Subsequent photo-activation of leaves did not change the pattern of the difference spectra (photo-activated – minus – control) (Figure 4D).

The difference FTIR spectra (Figure 4E) for bean thylakoids revealed more evident changes than in pea. The positive band around 1620 cm^–1^ was observed for both thylakoids isolated from chilled and subsequent photo-activated leaves (Figure 4E), suggesting noticeable changes in lamellar interactions between proteins.

### Composition of Lipids Phase of Bean and Pea Thylakoids Under Control and Stress Conditions

**FIGURE 5:**
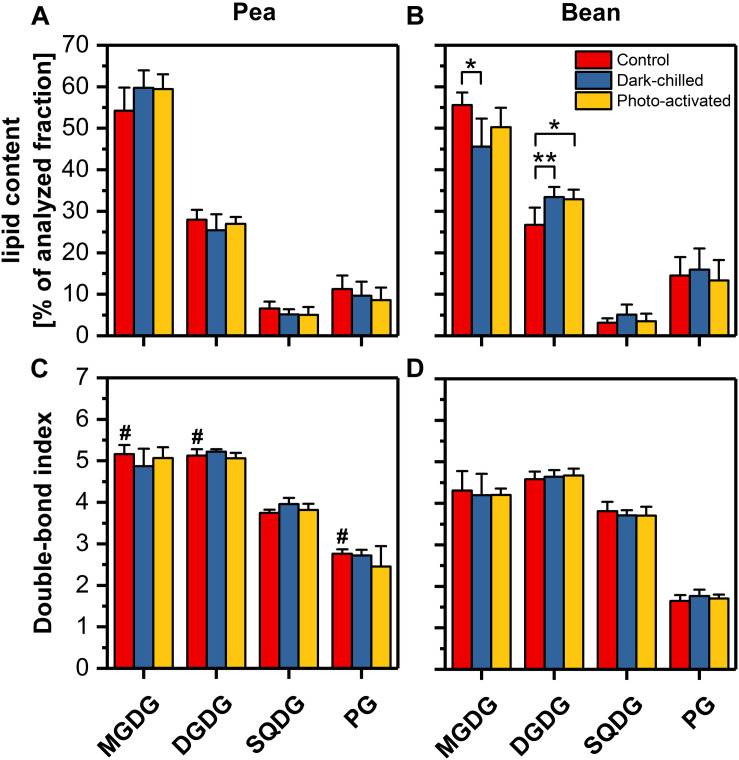
Profiles of the main four classes of lipids extracted from thylakoids isolated from pea **(A)** and bean **(B)** control (red), dark-chilled (blue) and subsequent photo-activation (orange) leaves. Lower panels present the double bond index (DBI) for corresponding lipid classes of pea **(C)** and bean **(D)** thylakoids. The data are mean values ± SD for 4–5 independent experiments. The indicating pairs of bars marked with an asterisk differ significantly at *p* = 0.05 (**) or 0.1 (*). Values marked with a hash indicate a significant difference (*p* = 0.05) between species in control samples.

The influence of lipid composition on pea and bean response to chilling conditions was analyzed by the comparison of the main four classes of thylakoid lipids (MGDG, DGDG, SQDG, and PG) (Figure 5). The control percentage contribution of main galactolipids MGDG and DGDG and their ratio were similar in both species (Figures 5A,B and Supplementary Table 2), but the abundance of anionic lipids, SQDG and PG significantly differed. The content of SQDG in pea control thylakoids was two-times larger, while the PG level was about three-times smaller than in bean (Figures 5A,B). The overall content of SQDG and PG constituted roughly 17% of the analyzed lipid fraction of the thylakoids of both species, but the SQDG to PG ratio was over two times higher in pea than in bean thylakoids (Supplementary Table 2).

The double-bond index (DBI) indicates the average number of double bonds in the fatty acid chains of a lipid molecular species; higher values of DBI correspond to the increase in membrane fluidity (Zheng et al., 2016). The average DBI of the control thylakoid lipids of pea was around 20% higher than for bean thylakoid lipids (Supplementary Table 2), which resulted from the higher DBI of only three pea lipid classes – MGDG, DGDG and PG in particular (Figures 5C,D). Furthermore, the relative content of the high melting point PG (32:0 and 32:1 molecular species) was almost five times higher in bean than in pea thylakoids (Supplementary Table 2). The DBI values for SQDG were almost equal in both species (Figures 5C,D).

The average acyl chain length (ACL) is the second indicator of the physical properties of the membrane – longer chains of fatty acids are related to lower fluidity of membrane (Zheng et al., 2016). The average ACL and ACL for MGDG and DGDG lipid classes only slightly differed between species (Supplementary Table 2).

After the dark-chilling treatment, the relative content of galactolipids and the ratio of lipid classes changed significantly in bean thylakoids only (Figures 5A,B). The MGDG to DGDG ratio decreased to 65% of the value estimated for the control bean samples (Supplementary Table 2), and the relative MGDG level decreased by 10% (Figure 5B). Simultaneously, the slight increase of SQDG to PG was noted in dark-chilled bean thylakoids (Supplementary Table 2). However, both in pea and bean thylakoids, the DBI and ACL did not change under dark-chilling conditions (Figures 5C,D and Supplementary Table 2). The photo-activation did not significantly influence the lipid composition of both species thylakoid membranes compared with dark-chilling conditions (Figures 5A,B). Similarly, the DBI and ACL parameters remained unchanged (Figures 5C,D and Supplementary Table 2).

### Carotenoid Composition of Pea and Bean Thylakoids in Control, Under the Dark-Chilling Treatment, and After Photo-Activation

Carotenoids play different roles in the thylakoid membranes they can (i) participate in photochemical reactions and dissipate the excess of light energy, (ii) effectively quench the free radicals and (iii) modify the fluidity of the lipid phase (Domonkos et al., 2013). Therefore, the determination of the carotenoid composition is important in assessing the physical properties of thylakoid membranes.

**FIGURE 6:**
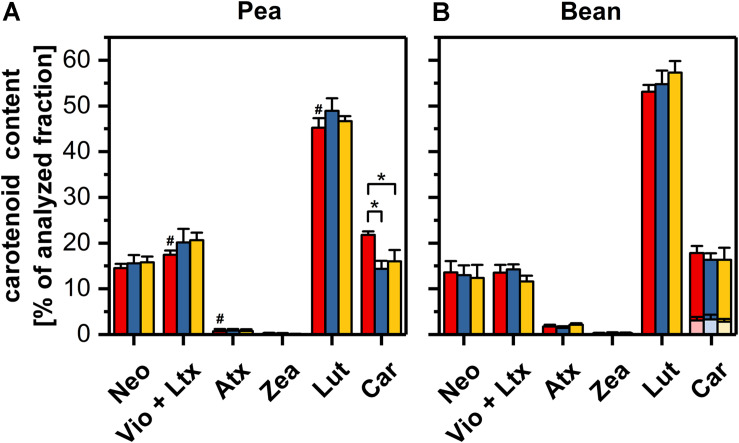
Carotenoid compositions of pea **(A)** and bean **(B)** thylakoids isolated from control (red), dark-chilled (blue) and subsequent photo-activation (orange) leaves. The profiles present the content of neoxanthin (Neo), a sum of violaxanthin and lutheoxanthin (Vio+Ltx), antheraxanthin (Atx), zeaxanthin (Zea), Lutein (Lut) and a sum of α- and β-carotene (Car). α- and β-carotene were labeled by transparent and opaque colors, respectively. The abundance of lutheoxanthin, which is converted non-enzymatically from violaxanthin did not exceed 1 mol % and therefore is presented as a sum with violaxanthin. The data are mean values ± SD for 5–8 independent experiments. Pairs of results marked with an asterisk differ significantly at *p* = 0.05. Values marked with a hash indicate a significant difference (*p* = 0.05) between species in control samples.

The content of the main carotenoids in control samples was significantly different in thylakoids from pea and bean leaves. In pea thylakoids, the lutein and β-carotene reached 45 and 2l% of the total carotenoid pool, respectively, making the lutein to β-carotene ratio equal 2. Bean thylakoids contained about 53% of lutein and 15% of β-carotene in the total carotenoid pool; the lutein/carotene ratio reached the value of almost 4 and the ratio of α- to β-carotene was estimated to 0.22 (Figures 6A,B and Supplementary Table 3). The relative content of neoxanthin was similar in both species, whereas the violaxanthin was slightly more abundant in pea thylakoids. The content of zeaxanthin and antheraxanthin did not exceed 0.2 and 1.7%, respectively (Figures 6A,B). The ratio of lutein to the sum of β-xanthophylls indicated noticeably higher value for bean thylakoids. Furthermore, the presence of α-carotene was noted in bean thylakoids only (Supplementary Table 3).

The exposure of leaves to dark-chilling conditions did not significantly affect the carotenoid compositions in both species except for a decrease of one-third of the β-carotene relative content in pea thylakoids (Figures 6A,B). The photo-activation of leaves of both species did not change the proportion of carotenoids in comparison with data obtained for dark-chilled leaves (Figures 6A,B).

### The Influence of Dark-Chilling on Pea and Bean Thylakoid Protein Patterns

**FIGURE 7:**
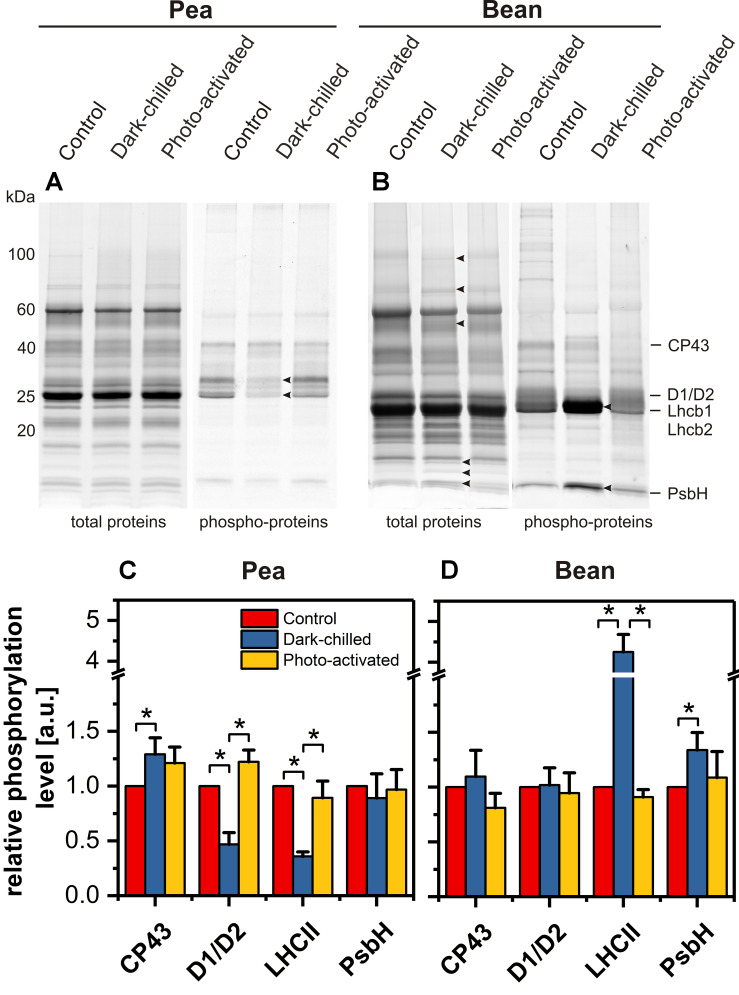
Changes of thylakoid protein and phospho-protein composition after dark-chilling and photo-activation in pea and bean plants. SDS-PAGE gels of pea **(A)** and bean **(B)** thylakoids stained with Sypro Ruby (protein) and Pro-Q Diamond (phospho-protein). The quantitative analysis of selected phospho-proteins of pea **(C)** and bean **(D)** thylakoids. Presented data are mean values ± SD from at least three independent experiments. Pairs of results marked with an asterisk differ significantly at *p* = 0.05.

The protein patterns of the thylakoid membrane fractions isolated from control, dark-chilled and photo-activated pea and bean leaves were analyzed using SDS-PAGE and fluorescence staining. In pea, there were no significant qualitative and quantitative changes in protein abundance in dark-chilled and photo-activated samples (Figure 7A, left panel). In contrast, in bean, dark-chilling induced multiple changes in the protein pattern of the thylakoid membranes (Figure 7B, left panel, arrowheads). Some of the proteins: lipoxygenase PCLOXA (96 kDa), Rubisco (55 and 15 kDa), and PsbQ (18 kDa) were identified in our previous study (Mazur et al., 2018).

The thylakoid protein phosphorylation pattern was established using Pro-Q Diamond staining of SDS-PAGE gels (Figures 7A,B right panels). Four protein groups were analyzed – proteins of PSII core (CP43, D1/D2, PsbH) and LHCII antennae (Lhcb1/Lhcb2). In thylakoids isolated from control pea leaves the relative phosphorylation levels of both antenna proteins and CP43 subunit were roughly 20% higher than in bean thylakoids, suggesting differences in the kinase activity or accessibility of substrates in both species.

In pea, dark-chilling induced a significant decrease in D1, D2, Lhcb1, and Lhcb2 phosphorylation and a slight increase of CP43 phosphorylation (Figure 7C) compared with control conditions. Such a change in thylakoid protein phosphorylation was typical for the thylakoids isolated from plants directly after the night period. In contrast, in bean, the dark-chilling induced 4-fold increase in the phosphorylation of LHCII major antenna proteins and 1.5-fold increase in PsbH protein phosphorylation (Figure 7D) compared with control conditions; the CP43 and D1/D2 phosphorylation levels were stable after chilling (Figure 7D). In both species, photo-activation of dark-chilled caused the return of protein phosphorylation status to the values of control samples (Figures 7C,D).

### Thylakoid Network Structure of Pea and Bean

**FIGURE 8:**
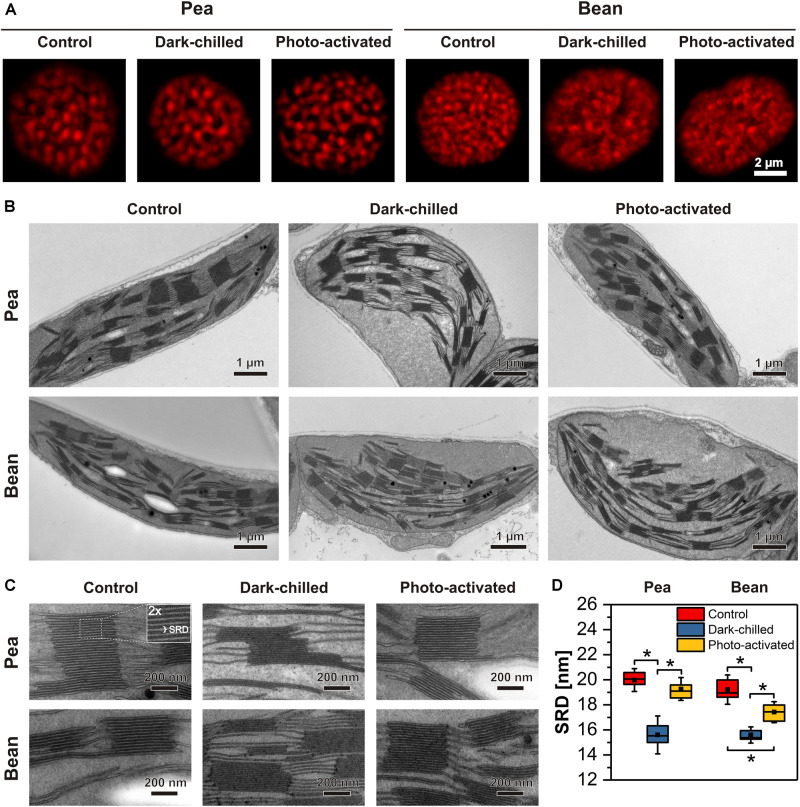
Structural changes of thylakoid network after dark-chilling and photo-activation in pea and bean plants. The images of intact chloroplasts visualized by confocal microscopy; red fluorescence spots roughly correspond to grana size and their position inside the chloroplast **(A)**. Electron micrographs of mesophyll chloroplasts **(B)** and grana enlargement showing changes in thylakoid network regularity and fluctuation in stacking repeat distance (SRD) **(C)**. The quantitative analysis of SRD values (*n* = 15–20 per each variant) **(D)**. The bottom and top of each chart box represent the 25 and 75 percentile, respectively, the whiskers denote the SD; pairs of results marked with an asterisk differ significantly at *p* = 0.05.

Details of the thylakoid network structure were analyzed using both CLSM, reveling the distribution of grana stacks and their organization within the whole chloroplast (Figure 8A), as well as TEM showing the detailed structure of stacked grana and unstacked stroma thylakoids (Figures 8B–D).

In the CLSM images, visible red fluorescence spots reflect mainly grana stacks containing LHCII trimers and LHCII-PSII supercomplexes. Therefore, the spot distribution corresponds to the position of grana stacks in the chloroplast (Rumak et al., 2010, 2012). In pea, large and well-distinguished spots were registered as opposed to smaller and more blurry fluorescence discs present in bean chloroplasts (Figure 8A). Such differences were even more profound after the dark-chilling treatment showing a disorganized thylakoid network in bean plants. In the case of pea, no significant changes in general features of the thylakoid network structure were registered (Figure 8A). In both species, photo-activation did not change the chloroplast fluorescence image significantly compared to the one registered after dark-chilling (Figure 8A).

More structural details were revealed by electron microscopy analysis of fixed leaf samples (Figures 8B,C). Chloroplast cross-sections of control samples showed a significant difference in grana distribution between both analyzed species. In pea, large grana stacks (432 ± 89 nm in diameter and 238 ± 122 nm in height) interconnected via stroma thylakoids were distributed parallel to each other and to the long chloroplast axis (Figure 8B). In the case of bean, grana stacks were of similar diameter (401 ± 79 nm) as in pea but with considerably smaller height (174 ± 101 nm). Moreover, the disturbance in such regular arrangement was visible, with multiple shifts of grana stacks position. After the dark-chilling treatment, swelling of the chloroplast stroma was registered in both examined species. After the photo-activation stroma swelling was visible in bean chloroplasts only (Figure 8B). Low temperature did not affect the pea thylakoid network organization visible at the level of the whole chloroplast section, while in bean, the thylakoid network disorganization proceeded continuously after dark-chilling and photo-activation (Figure 8B).

One of the important ultrastructural grana features is the degree of thylakoid stacking, expressed by stacking repeat distance (SRD), defined as the distance between adjacent partition gaps in the stacks (Kirchhoff et al., 2011; Figure 8C). In control plants, no significant differences in the SRD value (around 19–20 nm) were registered between both analyzed species (Figure 8D). Dark-chilling treatment induced a substantial decrease in the SRD value (15–16 nm) both in pea and bean. Although photo-activation caused an increase in the SRD value in both species, full recovery of grana stacking (SRD around 19 nm) was observed in pea grana only. In the case of bean, only partial SRD increase was observed, reaching values of around 17 nm.

## Discussion

The diversity of the chloroplast membrane network structure organized into stacked, marginal and unstacked regions is generally explained by the presence of a lateral heterogeneity of CP complexes and in consequence, different steric and physicochemical interaction between membranes (Jia et al., 2014; Garab, 2016; Koochak et al., 2019). The role of the lipid phase and lipid-protein interactions in the determination of the thylakoid structure is less explained (Garab et al., 2017). Moreover, the relationship between the stress-induced changes in the thylakoid structure and the changes in their protein or lipid composition, as well as the arrangement of the CP complexes is unclear. Since the lipid composition of thylakoids, especially the degree of thylakoid lipids desaturation, is related to plant sensitivity to chilling (Kenchanmane Raju et al., 2018), further studies should be applied to reveal the connection between plant sensitivity to low-temperature and the lipid-protein interactions in the thylakoids of CT and CS plants.

### Composition and Arrangement of CT Pea and CS Bean Thylakoid Membranes in Optimal Conditions – Background to Stress Response

We have analyzed the two plant species belonging to two separate groups due to their different responses to chilling conditions and revealing different thylakoid network structures. The observations with the use of TEM and CLSM showed that chloroplasts in pea contain larger stacked areas than in bean, in which the stacked regions are less distinguished (Figure 8). It was established before that observed ultrastructural differences between both species depend on the diversity of the thylakoid protein composition and arrangement, and in consequence, in different protein-protein interactions (Rumak et al., 2012).

The observed differences in the thylakoid structure and the arrangement of the CP complexes might be, moreover, partially explained by the particular lipid composition and lipid-protein interactions of the thylakoid membrane matrix of CT pea and CS bean plants analyzed in this study. Analysis of lipid profiles revealed that the MGDG and DGDG constitute more than 80% of total lipids, and their ratio is similar in pea and bean (Figure 5 and Supplementary Table 2). Despite the similarities in the content of neutral lipids between both analyzed species, the lipid/protein ratio in pea thylakoids is noticeably higher than in bean (Figure 4). These data, together with the specific macro-domain organization of LHCII-PSII in both species (Rumak et al., 2012), point to the higher amount of lipids in the bulk phase of pea than of bean membranes and thus presumably increased membrane fluidity in this species.

Moreover, the SQDG to PG ratio is two times higher in pea than in bean thylakoids. However, the sum of these anionic lipids is similar in both analyzed species (Figure 5 and Supplementary Table 2). Such results are in line with studies on lipid deficient plants pointing to the importance of the maintenance of the sum of the anionic lipids in thylakoid network formation and fitness (Yu and Benning, 2003; Kobayashi et al., 2017).

Another factor influencing thylakoid membrane fluidity is the degree of thylakoid lipid desaturation. Polyunsaturated fatty acids building acyl chain of galacto- and phospholipids stabilize the liquid-crystalline phase of the membrane. The average DBI is roughly 20% higher for pea than for bean thylakoid lipids with significant differences between lipid classes. The DBI for PG is 60% higher in pea than in bean (Figure 5) – PG desaturation level is frequently attributed to increased chilling tolerance (Ivanov et al., 2012).

Moreover, the 32:0 and 32:1 PG molecules are high-melting-point molecular species which, under the *in vitro* conditions, undergo the liquid-crystalline to gel phase transition at room temperature i.e., induce the rigidification of membranes. This effect is not directly observed in the thylakoid membranes because of the predominant abundance of desaturated lipids. However, the positive correlation between the amount of these PG species and sensitivity to low-temperature was found in a wide variety of plants and transgenic lines (Szalontai et al., 2003; Los et al., 2013). For example, CT Arabidopsis contains two and eight times lower amounts of the 32:0 and 32:1 PG species than CS rice (Zheng et al., 2016). Similarly, our data show five times lower abundance of high-melting-point PG species in CT pea than in CS bean thylakoids (Supplementary Table 2). These data indicate higher fluidity of the thylakoid membranes in CT pea compared with CS bean and agree with the observation that the higher desaturation level of lipids is correlated with the higher resistance to chilling (Los et al., 2013; Zheng et al., 2016; Kenchanmane Raju et al., 2018).

The lower value of the ACL of the total lipid pool correlates with the higher fluidity of the thylakoid membranes (Zheng et al., 2016). The ACL for MGDG and DGDG is slightly higher in pea thylakoids than in bean and their values do not change during chilling treatment (Supplementary Table 2), which indicates no simple correlation between the ACL values and the resistance to chilling.

Chloroplast lipid metabolism involves the activity of many types of deacylating enzymes (Matos and Pham-Thi, 2009). Significantly higher activity of galactolipase in CS than in CT species was reported previously (Kaniuga, 2008). The galactolipase isolated from bean chloroplasts had almost ten times higher activity compared with pea one (Gemel and Kaniuga, 1987), and these activities were associated with two-times higher accumulation of FFA registered in bean chloroplasts (Gemel and Kaniuga, 1987; Garstka et al., 1994). The accumulated FFA might influence the structure of thylakoids, however, the FFA undergo enzymatic and non-enzymatic peroxidation, which might decrease its detergent-like effect (Garstka et al., 1994; Mazur et al., 2018).

Free hydrophobic carotenoids, not bound to proteins, are embedded in membranes and can modify the physical properties of the lipid bilayer. Xanthophylls that contain polar groups at the two ends of the molecule and are positioned across the bilayer, cause the rigidification of membranes. On the other hand, the β-carotene embedded regardless of the orientation type within the membrane increases its fluidity. Thus, in addition to the desaturation level of lipids, the balance between xanthophylls and β-carotene helps to maintain the optimal fluidity of thylakoid membranes under the temperature stress (Gruszecki and Strzalka, 2005; Szilagyi et al., 2008; Domonkos et al., 2013). The bean thylakoids revealed a significantly higher ratio between main xanthophyll – lutein and the β-carotene, pointing to the lower fluidity of bean membranes under control conditions compared with pea (Figure 6 and Supplementary Table 3). Furthermore, both the higher ratio of lutein to the sum of β-xanthophylls and the presence of α-carotene in bean thylakoids (Supplementary Table 3), suggest a higher activity of the α-xanthophyll branch of the carotenoid biosynthetic pathway in bean than in pea chloroplasts (Domonkos et al., 2013).

Apart from the lipids forming the thylakoid membrane matrix, lipids are bound inside the protein scaffold of supercomplexes playing an important role in the stabilization of their structure and maintaining their photochemical functions (Jones, 2007; Domonkos et al., 2008). Based on the crystallography analyses, the abundance of integral lipids in LHCII-PSII and LHCI-PSI supercomplexes is estimated to 4.6 and 3.7% of the total pool of thylakoid lipids, respectively. Interestingly, almost 30% of the PG pool is associated with CP complexes, LHCII-PSII in particular (Kobayashi et al., 2017). Many investigations indicated that more than 50% of lipids in grana thylakoids are localized in the lipid-protein interface (Pali et al., 2003), and the “molecular dynamic simulation” showed that annular shell around PSII dimer is selectively enriched with MGDG and SQDG (Van Eerden et al., 2017). Such an ordered phase is probably larger than the bulk phase which contains lipids with higher fluidity (Azadi Chegeni et al., 2016). Therefore, it might be possible that maintaining the optimal fluidity of thylakoids depends more on lipid-protein interactions than lipid composition alone.

The relationship between the supramolecular membrane structure and the photochemical reactions can be analyzed by temperature dependencies of Chl *a* fluorescence emission measured in F_0_ or steady-state (Wentworth et al., 2003; Tovuu et al., 2013; Zubik et al., 2013). The breakpoint in the linear temperature-dependent plot indicates the changes in the interactions between CP complexes due to a temperature-induced structural transformation. The temperature-dependent plot of Chl fluorescence for thylakoids isolated from control pea and bean leaves differ in the number of breakpoints; two and three phases of the fluorescence decrease in pea and bean, respectively (Figures 2A,D). The second and the third phase of the fluorescence decrease in bean revealed a similar slope as analogous phases for pea (Supplementary Table 1), indicating the similar interactions needed for the rearrangement of CP complexes.

The temperature-dependent changes in the fluorescence decrease arise from small changes in the conformation of CP complexes. Such changes may comprise alterations in the hydrogen and van der Waals interactions induced both by protein-protein and lipid-protein interactions. The breakpoint of the temperature-dependent plot might be correlated with a transition temperature of the lipid phase (Kovacs et al., 2019). Our data (Figure 2) showed that fluidity of bean thylakoid membranes is lower than in pea what indicates that at low temperatures, the possibility of gel phase formation is higher in bean thylakoids. Therefore, we propose that the one-breakpoint plot for pea thylakoids might be attributed to the phase transition between liquid-crystalline and disorder phases, whereas the two-breakpoint plot for bean thylakoids is related to the transition from gel-phase to liquid-crystalline and further to disorder phase (Los et al., 2013).

Species-dependent regulation of the thylakoid membrane fluidity is considered as an evolutionary adaptation mechanism to cope with high or low-temperature stress (Zheng et al., 2016; Kenchanmane Raju et al., 2018). The efficiency of photochemical reactions, among different factors, is regulated by the mobility of the electron transport chain components in the lateral plane of thylakoid membranes. To maintain appropriate transport within the lipid matrix in low temperatures its fluidity has to be preserved. Our results showed that the supramolecular organization of CP complexes, lipid composition and DBI index, lutein/β-carotene and protein/lipid ratios differ between CT pea and CS bean, indicating higher fluidity of the thylakoid membrane network in pea in optimal temperature conditions.

### Effects of Dark-Chilling and Subsequent Photo-Activation on Composition and Arrangement of Thylakoid Membranes

Under dark-chilling and subsequent photo-activation at moderate light both in pea and bean, there is no decrease of the chlorophyll amount, no changes in Chl *a* to Chl *b* ratio (Garstka et al., 2005, 2007), and direct fluorescence emission from Chl *b* molecules in thylakoid samples is not observed (Figure 3). These data indicate that CP complexes are not degraded under applied experimental conditions. However, data obtained by mild-denaturing electrophoresis (Garstka et al., 2005, 2007) and spectroscopic measurements (Figures 3, 4), revealed that under the dark-chilling conditions the arrangement of CP complexes is significantly affected in bean but not in pea thylakoids. Therefore, the lack of significant changes in the photochemical parameters and the course of the fluorescence induction curves (Figure 1) can be directly related to the stable behavior of pea supercomplexes under dark-chilling and subsequent photo-activation (Figures 3, 4). In contrast, a decrease of F_V_/F_M_ value, the change in the course of the fluorescence induction curves, as well as the lowered capacity of intersystem electron carrier pool (Figure 1) in bean thylakoids correlate with significant changes in the arrangement of CP complexes (Figures 3, 4) and the increase of LHCII phosphorylation (Figure 7). These reorganizations of CP complexes result to some extent in LHCII-dependent energy spillover (Figures 3, 4). Moreover, the presence of phosphorylated LHCII pool causes the increase of the negative charge of the stromal side of the thylakoid membrane, which changes the balance between the attractive and repulsion forces between neighboring CP complexes, and therefore alters their supramolecular organization (Puthiyaveetil et al., 2017). Furthermore, only the partial recovery of the bean photochemical activity after photo-activation (Figure 1) can be related to incomplete restoration of the native structure of CP complexes (Figures 3, 4) manifested mainly by the appearance of the aggregated LHCII (Figure 3).

Dark-chilling treatment only slightly influences the lipid phase of pea thylakoids, both in terms of values of DBI and ACL, as well as lipid composition in which some increase in the MGDG/DGDG ratio under dark-chilling is observed (Figure 5 and Supplementary Table 2). Xanthophyll relative content does not change significantly, but a decline in the relative level of β-carotene is observed (Figure 6), probably due to the antioxidant role of β-carotene inactivating reactive oxygen species present in stress conditions (Domonkos et al., 2013). This change is also reflected in a decrease of the lutein/β-carotene ratio (Supplementary Table 3), attributed to the increase of membrane fluidity (Gruszecki and Strzalka, 2005; Szilagyi et al., 2008). However, in pea, the overall fluidity of thylakoid membranes remains similar to the control conditions, which is visible in the same course of the temperature-dependent plots for the control and dark-chilling plants (Figures 2A–C and Supplementary Table 1). Probably, the high level of lipid desaturation in control pea (Figure 5C) retains the optimal fluidity at low temperatures and prevents the loss of the CP complexes functionality in dark-chilled plants (Figure 1).

In contrast, the substantial changes in lipid composition are observed after dark-chilling of bean leaves. The significant decline in the MGDG and increase in the DGDG levels cause a 35% decrease in the MGDG/DGDG ratio; SQDG and PG levels remained unchanged (Figure 5B and Supplementary Table 2). In various plant species, the long-term cold-adaptation includes a decrease in the MGDG level, probably because lower level of the MGDG stabilizes the membrane bilayer phase at low temperatures (Zheng et al., 2016; Kenchanmane Raju et al., 2018).

Simultaneously, the rigidity of membranes induced by low-temperature is alleviated by the increase in the DBI index and a decrease in the ACL index (Zheng et al., 2016). It was previously proposed that the thylakoid lipids of the CT plants remain in the liquid-crystalline phase whereas thylakoids in the CS species enter the gel phase at chilling temperatures, mainly due to different levels of lipid desaturation (Szalontai et al., 2003; Los et al., 2013; Kenchanmane Raju et al., 2018). The level of lipid saturation is related to the activity of desaturases and transferases regulated directly or non-directly by factors connected with the C-repeat binding factor signaling pathway (Thomashow, 2010; Kenchanmane Raju et al., 2018). In thylakoids isolated from dark-chilled bean leaves the DBI and ACL indexes are maintained at the same level as in control thylakoids (Figure 5 and Supplementary Table 2), which exclude activation of typical long-term adaptation processes during low-temperature treatment in bean.

Dark-chilling-induced changes in bean thylakoids, in lipid composition in particular, substantially alters the membrane properties, which is reflected by a drastic change in the course of the temperature-dependent plot; the three-phase plot is converted to a single-phase one (Figures 2D,E). The temperature-dependent plot without breakpoint indicates impairment of the lipid-protein interactions or lack of lipid phase transition in the measured temperature range. It is probably related to the detergent-like effect caused by the accumulation of FFA in thylakoids, which is typical for CS plants under dark-chilling stress (Kaniuga, 2008). Under these conditions, the FFA level in bean thylakoids increases two times and remains unchanged in pea (Garstka et al., 1994). Such variable response is correlated with higher activity of galactolipase in bean than in pea plants (Gemel and Kaniuga, 1987). Photo-activation of dark-chilled leaves of CS plants results in a decrease of FFA to the level observed in control plants (Garstka and Kaniuga, 1991; Kaniuga, 2008) probably due to the increase of peroxidative reactions (Garstka et al., 1994). This effect might be a reason why in photo-activated bean thylakoids we observed the restoration of the three-phase temperature-dependent plot with breakpoints characteristic for the control thylakoids (Figures 2D,F) without significant changes in the ratios of lipids and carotenoids (Figures 5, 6). Moreover, changes in membrane properties of bean thylakoids during dark-chilling and photo-activation explain the reversible association of stromal proteins Rubisco and PcLOXA lipoxygenase (Figure 7; Mazur et al., 2018).

In contrast to pea, dark-chilling induces significant alternation in the lipid phase, thylakoid protein phosphorylation status, and arrangement of CP complexes in bean (Figures 3, 5, 7) which result in increased disorganization of the thylakoid network visible at the ultrastructural level (Figure 8). The photo-activation of bean leaves considerably restores the physical properties of membranes (Figure 2) and partially the structure and photochemical activity of CP complexes (Figures 1, 3, 7). However, the disorganized arrangement of the thylakoid network is still visible (Figure 8). Experiments with Mg^2+^-induced stacking of thylakoids revealed that the protein diffusion in bean thylakoids is limited due to spatial encumbrance caused by the heterogeneous arrangement of CP complexes (Rumak et al., 2010). Such a specific supramolecular thylakoid structure in combination with the aggregation of LHCII induced by photo-activation (Figures 3, 4) makes a return to initial thylakoids organization difficult.

One of the important factors that influence the thylakoid grana ultrastructure is the reversible phosphorylation of PSII proteins, LHCII antenna in particular. LHCII phosphorylation causes partial unfolding of the granum structure (reviewed in Kirchhoff, 2019). The SRD value, which is one of the parameters describing the extent of grana stacking, decreases in dark-chilled pea thylakoids which is correlated with a decrease of LHCII phosphorylation (Figures 7, 8). Despite a substantial increase in LHCII phosphorylation in dark-chilled bean thylakoids, a decrease of the SRD value is observed. This indicates that there is no simple correlation between grana stacking and LHCII phosphorylation level in dark-chilling conditions. Under dark-chilling conditions, the bean thylakoid network comprises multiple small grana connected via many stroma lamellae (Figures 8B,C; Rumak et al., 2012), which causes the increase of the total size of the marginal regions of the grana thylakoids and probably also the access of LHCII for the phosphorylation.

The phosphorylation of the LHCII in the darkness was described before and might be attributed to the non-photochemical PQ reduction (Nellaepalli et al., 2012a, b). The PQ reduction is essential to the activation of STN7 kinase for which LHCII is a primary target. However, the possibility of the phosphorylation of LHCII via other kinases was proposed recently (Longoni et al., 2019) and their activity in dark-chilling conditions cannot be excluded.

## Conclusion

The direct and immediate effect of low temperature on the physical properties of the membrane is related to a decrease in mobility of the acyl chains and their stiffness. It induces activation of response mechanisms that are different in chilling sensitive and chilling tolerant plants. The direction and magnitude of this response depend on the evolutionary background of the species.

In this study, we revealed that the different susceptibility of CS bean and CT pea plants to dark-chilling treatment is mainly attributed to a particular, species-dependent, composition of their thylakoid lipid phases, manifested by specific DBI level, saturated PG content and protein/lipid ratio. The composition of the thylakoid lipid matrix of CT pea allows retaining the optimal fluidity of its chloroplast membranes under low temperatures. In contrast, the fluidity of CS bean thylakoids is drastically changed under the dark-chilling treatment which is the result of MGDG hydrolysis and in consequence, accumulation of FFA. Changes in lipid matrix properties leading to the reorganization of the supramolecular structure of photosynthetic complexes finally cause structural remodeling of the CS bean thylakoid network in dark-chilling conditions.

## Data Availability Statement

The datasets generated for this study are available on request to the corresponding author.

## Author Contributions

MG, RM, and KG provided conception of the manuscript. RM, KG, AK, MP, ŁK, and MG performed the experiments. MG, RM, ŁK, and AM wrote the manuscript. All authors read and approved the final manuscript, designed the experiments, and analyzed the data.

## Conflict of Interest

The authors declare that the research was conducted in the absence of any commercial or financial relationships that could be construed as a potential conflict of interest.
