# Supplementary material for: Specific Composition of Lipid Phases Allows Retaining an Optimal Thylakoid Membrane Fluidity in Plant Response to Low-Temperature Treatment
Source: Front Plant Sci. 2020 Jun 5;11:723. doi: 10.3389/fpls.2020.00723 (PMC7291772; doi:10.3389/fpls.2020.00723)
Supplement: Supplementary file 1 [file Data_Sheet_1.PDF]

SUPPLEMENTARY MATERIAL

Specific Composition of Lipid Phases Allows Retaining an Optimal Thylakoid Membrane Fluidity in Plant Response to Low-Temperature Treatment

Radosław Mazur[1#*], Katarzyna Gieczewska[2#], Łucja Kowalewska[2], Anna Kuta[1$], Małgorzata Proboszcz[1§], Wieslaw I. Gruszecki[3], Agnieszka Mostowska[2], Maciej Garstka[1*]

[1] Department of Metabolic Regulation, Institute of Biochemistry, Faculty of Biology, University of Warsaw, Miecznikowa 1, 02-096 Warsaw, Poland
[2] Department of Plant Anatomy and Cytology, Institute of Plant Experimental Biology and Biotechnology, Faculty of Biology, University of Warsaw, Miecznikowa 1, 02-096 Warsaw, Poland
[3] Department of Biophysics, Institute of Physics, Maria Curie-Skłodowska University, 20-031 Lublin, Poland

Correspondence:
Prof. Maciej Garstka
garstka@biol.uw.edu.pl

Dr Radosław Mazur
rmazur@biol.uw.edu.pl

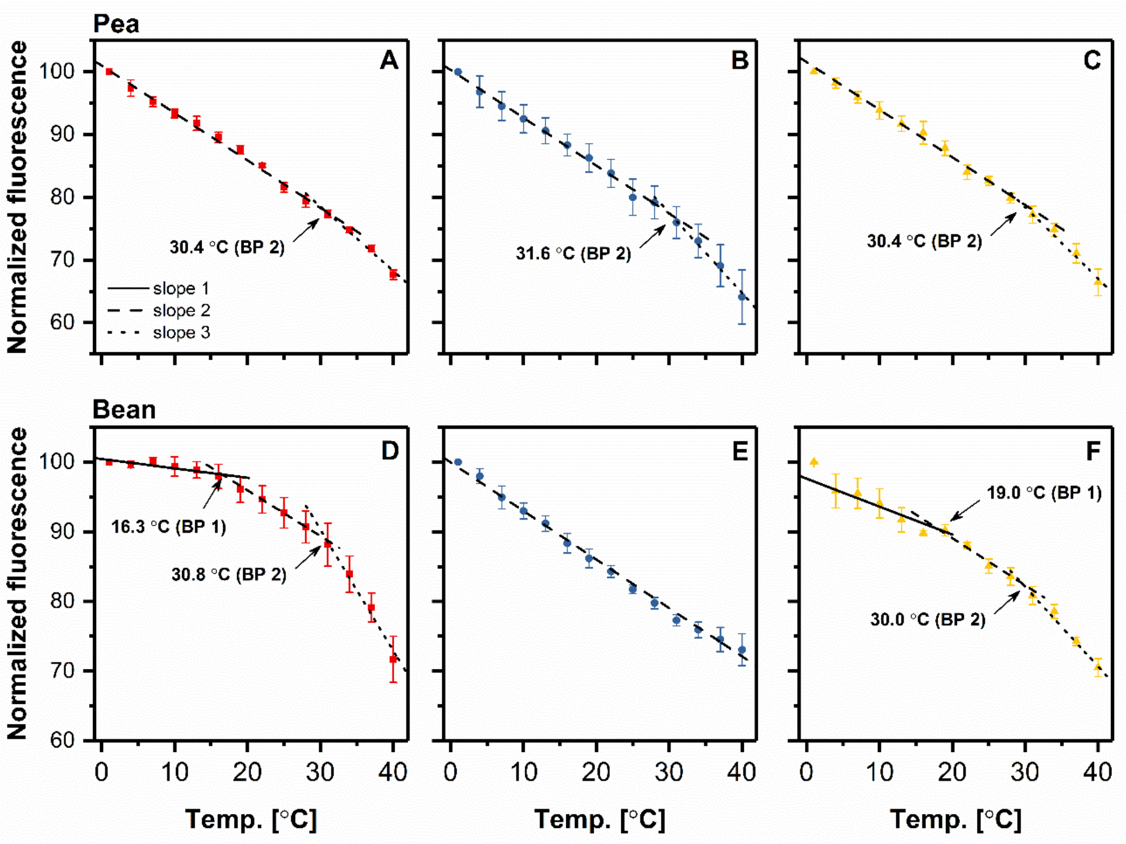

Supplementary Figure 1.

Temperature-dependent plots of the rate of chlorophyll fluorescence decrease in pea (A-C) and bean (D-F) thylakoids isolated from control (A, D), dark-chilling (B, E) and subsequent photo-activated (C, F) leaves. Fluorescence emission at 680 nm was excited at 412 nm. The data are mean values ± SD for 3 to 5 independent experiments. The values for breakpoints (BP) were calculated by linear regression independently for each experiment. For a clear presentation of plots, the two from three consecutive points were omitted.

Supplementary Table 1.

Calculated breakpoints and fluorescence decrease rates for the temperature-dependent plots of fluorescence decrease for thylakoids isolated from control, dark-chilled and subsequently photo-activated pea and bean leaves

	Pea			Bean		
	Control	Dark-chilled	Photo-activated	Control	Dark-chilled	Photo-activated
Estimated breakpoint between fluorescence decrease phases [°C]						
Ex 412 nm						
BP1	-	-	-	16.3 ± 0.9	-	19.0 ± 2.8
BP2	30.4 ± 1.6	31.6 ± 0.6	30.4 ± 0.6	30.8 ± 0.3	-	30.0 ± 1.6
Ex 470 nm						
BP1	-	-	-	17.2 ± 0.4	-	18.4 ± 1.1
BP2	29.9 ± 1.0	31.1 ± 0.3	30.2 ± 0.8	30.4 ± 0.4	-	30.7 ± 1.1
Estimated rate of fluorescence decrease [Δ Flu /°C]						
Ex 412 nm						
Slope 1	-	-	-	0.20 ± 0.10	-	0.44 ± 0.17
Slope 2	0.76 ± 0.05	0.77 ± 0.08	0.75 ± 0.04	0.65 ± 0.11	0.71 ± 0.08	0.67 ± 0.03
Slope 3	1.06 ± 0.06	1.48 ± 0.58	1.18 ± 0.20	1.72 ± 0.46	-	1.16 ± 0.10
Ex 470 nm						
Slope 1	-	-	-	0.27 ± 0.12	-	0.36 ± 0.14
Slope 2	0.69 ± 0.05	0.69 ± 0.05	0.69 ± 0.05	0.72 ± 0.06	0.70 ± 0.07	0.77 ± 0.14
Slope 3	1.01 ± 0.07	1.31 ± 0.40	1.09 ± 0.14	1.41 ± 0.08	-	1.66 ± 0.48

The values for breakpoints were calculated by linear regression as a cross point of two consecutive lines and the rates of fluorescence decease were equal to degree of slope estimated by linear regression for appropriate linear phase. Values were calculated independently for each experiment and the data are mean values ± SD (n = 3 – 5).

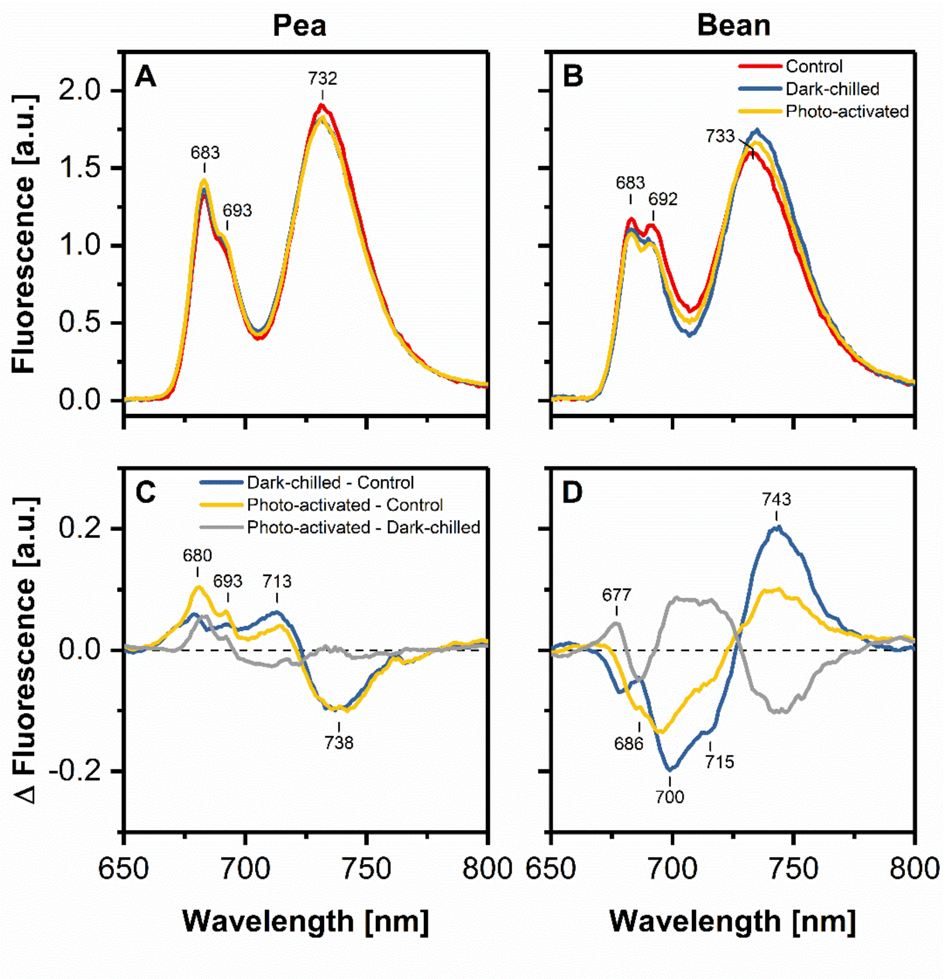

Supplementary Figure 2.

Effect of dark-chilling and subsequent photo-activation on fluorescence emission spectra (Ex 412 nm) at 77 K of isolated thylakoids from control (red), dark-chilled (blue) and subsequently photo-activated (orange) leaves of pea (A) and bean (B), respectively. Lower panels present the corresponding difference fluorescence spectra for pea (C) and bean (D). The spectra (A, B) were normalized to the area of 100 under the spectrum, and the arithmetic differences (C, D) between them were calculated. The presented spectra are representative of 3 – 5 independent experiments.

Supplementary Table 2.

Characteristic of lipid classes in thylakoids isolated from control, dark-chilled and subsequent photo-activated leaves of pea and bean plants

	Pea			Bean		
	Control	Dark-chilled	Photo-activated	Control	Dark-chilled	Photo-activated
				Ratio of lipid classes		
MGDG/DGDG	1.96 ± 0.29	2.41 ± 0.46	2.21 ± 0.10	2.12 ± 0.29[a]	1.37 ± 0.18[b]	1.53 ± 0.07[ab]
SQGDG/PG	0.59 ± 0.04[#]	0.55 ± 0.09	0.58 ± 0.09	0.22 ± 0.03	0.31 ± 0.06	0.25 ± 0.04
32:0 PG/ sum of PG	0.06 ± 0.01[#]	0.06 ± 0.01	0.06 ±0.01	0.26 ± 04	0.25 ± 0.03	0.24 ±0.03
32:1 PG/ sum of PG	0.05 ± 0.01[#]	0.04 ± 0.00	0.04 ±0.00	0.26 ±0.04	0.28 ± 0.01	0.29 ± 0.04
				Double-bond index (DBI)		
Average DBI	4.80 ± 0.20[#]	4.71 ± 0.28	4.79 ± 0.14	3.98 ± 0.35	3.94 ± 0.32	4.00 ± 0.12
				Acyl chain length (ACL)		
Average ACL	35.41 ± 0.09[#]	35.40 ± 0.10	35.45 ± 0.05	34.93 ± 0.18	34.86 ± 0.17	34.91 ± 0.11
MGDG	35.73 ± 0.06[#]	35.60 ± 0.14	35.69 ± 0.09	35.33 ± 0.18	35.25 ± 0.19	35.27 ± 0.04
DGDG	35.50 ± 0.05[#]	35.57 ± 0.10	35.50 ± 0.04	35.21 ± 0.08	35.25 ± 0.02	35.21 ± 0.06
SQDG	34.33 ± 0.04	34.31 ± 0.04	34.29 ± 0.05	34.34 ± 0.09[a]	34.53 ± 0.09[b]	34.41 ± 0.03[ab]
PG	34.19 ± 0.10	34.28± 0.07	32.23 ± 0.11	33.04 ± 0.14	32.98 ± 0.07	32.95 ± 0.08

The DBI and ACL are calculated as sum of percentage participation of total number of double bonds (N) or total number of carbons (n) in the two fatty acid chains of each lipid molecular species or of all identified lipids, according to equation DBI(ACL)=Σ[N(n) × % lipid])/100 (Zheng et al. 2016)

Values marked with a hash indicate significant difference (p = 0.05) between species in control samples. Values denoted by different letters significant difference (p = 0.05) between experimental conditions inside single species.

Supplementary Table 3.

The ratios of carotenoid species extracted from thylakoids isolated from control, dark-chilled and subsequent photo-activated leaves of pea and bean plants

	Pea			Bean		
	Control	Dark -chilled	Photo-activated	Control	Dark -chilled	Photo-activated
			Ratio of carotenoids species			
Lutein/ β-carotene	2.08 ± 0.15[#]	3.49 ± 0.45	3.01 ± 0.55	3.89 ± 0.27[a]	4.02 ± 0.51[ab]	4.17 ± 1.16[b]
α-carotene/ β-carotene	-	-	-	0.22 ± 0.07	0.26 ± 0.09	0.22 ± 0.06
Lutein/ sum of β-xanthophylls	1.39 ± 0.07	1.23 ± 0.25	1.26 ± 0.09	1.84 ± 0.14	1.94 ± 0.30	2.20 ± 0.25

The carotenoids ratio were calculated independently for each experiments and average value ± SD were calculated from 5 to 8 independent experiments. Sum of β-xanthophylls is equal to the sum of neoxanthin + violaxanthin + lutheoxanthin + antheraxanthin + zeaxanthin. Values marked with a hash indicate significant difference (p = 0.05) between species in control samples. Values denoted by different letters significant difference (p = 0.05) between experimental conditions inside single species.
